# An Automatic Surface Defect Inspection System for Automobiles Using Machine Vision Methods

**DOI:** 10.3390/s19030644

**Published:** 2019-02-04

**Authors:** Qinbang Zhou, Renwen Chen, Bin Huang, Chuan Liu, Jie Yu, Xiaoqing Yu

**Affiliations:** 1 State Key Laboratory of Mechanics and Control of Mechanical Structures, College of Aerospace Engineering, Nanjing University of Aeronautics and Astronautics, Nanjing 210016, China; zhouqinbang@nuaa.edu.cn (Q.Z.); binhuang@nuaa.edu.cn (B.H.); chuanliu@nuaa.edu.cn (C.L.); 2 COMAC ShangHai Aircraft Design and Research Institute, Shanghai 201210, China; yujie1@comac.cc; 3 China National Aeronautical Ratio Electronics Research Institute, Shanghai 200241, China; yuxiaoqing_tl@163.com

**Keywords:** flaw detection, automatic visual inspection, machine vision, feature extraction, support vector machine (SVM)

## Abstract

Automobile surface defects like scratches or dents occur during the process of manufacturing and cross-border transportation. This will affect consumers’ first impression and the service life of the car itself. In most worldwide automobile industries, the inspection process is mainly performed by human vision, which is unstable and insufficient. The combination of artificial intelligence and the automobile industry shows promise nowadays. However, it is a challenge to inspect such defects in a computer system because of imbalanced illumination, specular highlight reflection, various reflection modes and limited defect features. This paper presents the design and implementation of a novel automatic inspection system (AIS) for automobile surface defects which are the located in or close to style lines, edges and handles. The system consists of image acquisition and image processing devices, operating in a closed environment and noncontact way with four LED light sources. Specifically, we use five plane-array Charge Coupled Device (CCD) cameras to collect images of the five sides of the automobile synchronously. Then the AIS extracts candidate defect regions from the vehicle body image by a multi-scale Hessian matrix fusion method. Finally, candidate defect regions are classified into pseudo-defects, dents and scratches by feature extraction (shape, size, statistics and divergence features) and a support vector machine algorithm. Experimental results demonstrate that automatic inspection system can effectively reduce false detection of pseudo-defects produced by image noise and achieve accuracies of 95.6% in dent defects and 97.1% in scratch defects, which is suitable for customs inspection of imported vehicles.

## 1. Introduction

The external appearance of a vehicle is of outmost importance as it gives consumers their first impression [1]. However, due to the existence or occurrence of surface defects (small scratches or dents) during the process of manufacturing and cross-border transportation of imported vehicles which can bring huge economic losses to automobile import agency companies and imported vehicle buyers, automobile agency companies will arrange for professional human checkers [2] to conduct pipelined manual inspections of imported vehicles, even for minor defects (up to 0.5 mm in diameter), after each shipment. Unqualified vehicles with surface defects are picked up to be returned to the factory for repair. The traditional checking procedure is a random sampling method of manual inspection in the product pipeline, judging whether the product is qualified or not by observing the differences of the surface appearance of the target product with the eyes, and eliminating the unqualified product manually. This detection method cannot meet the needs of modern industrial production, because the current manual inspection process has the following issues:

- The criteria for human vision are not quantified, but more dependent on subjective evaluation, so the criteria for each checker judging the product quality are not the same.
- Due to the large output of the product and the large number of inspection items, the inspector will experience ocular fatigue on the assembly line due to the high-intensity repetitive work which leads to less reliable defect detection making the quality of product not fully guaranteed.
- When the surface defect is not obvious, the help of external conditions (such as strong lighting environment) is needed to detect it. It is difficult to easily identify the surface defects only by the human eye, and it is impossible to achieve a continuous and stable workflow, resulting in greatly reduced production efficiency.

Automatic visual inspection systems (AISs), however, have gained great popularity with the development of programmable hardware processors and built-in image processing techniques which have the characteristics of fast speed, high precision, low cost and nondestructive features, improve productivity, quality management and provide a competitive advantage to industries that employ this technology [3]. This paper focuses on the core techniques of AIS.

**Figure 1:**
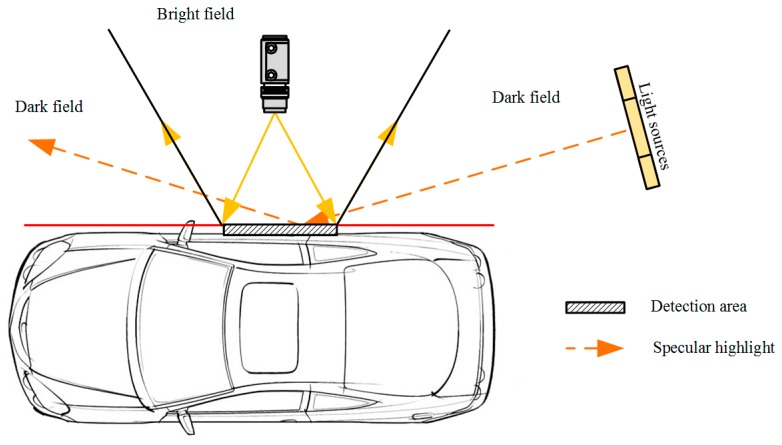
Sketch of dark field illumination.

Vehicle surface defects are broadly divided into two categories: scratches or dents. A scratch is a defect that occurs when a vehicle rubs against a hard raised metal during loading into a carrier or container transportation. The dents on the vehicle body are usually caused by the small stones that are propelled from the road surface hitting the surface of the vehicle traveling at a high speed. These defects are difficult to detect with automatic visual inspection systems (and even for human eyes) because of various factors:

- Imbalanced illumination: Vehicle surface images are captured by cameras installed at slide rails under the irradiation of four LED lights. The light mode determines that the vehicle body surface cannot achieve full uniform illumination, therefore global features or threshold parameters are not suitable for defect positioning and recognition.
- Specular highlight reflection: The dark field illumination technique (Figure 1) is utilized to enhance the contrast in defect samples. Even in the case of dark-field illumination, the captured vehicle frame gap may still cause specular highlights where the normal surface is oriented precisely halfway between the direction of incoming light (called half-angle direction because it bisects (divides into halves) the angle between the incoming light and the viewer).
- Variations of reflection modes for different vehicle colors: The surfaces of vehicle bodies do not share the same reflection modes. Using the comparison between a black vehicle body and a white vehicle body as an example, the gray-value of black vehicle defects present higher value than the body background, whereas the white vehicle shows lower values because of the diffuse reflection under different reflection coefficients.
- Limited features for defect recognition: Defects on the vehicle surface do not share common textures or shape features. Further, the pixel area of defects is even less than 20 pixels for tiny scratches or dents. The most reliable feature to distinguish surface defects is local gray-level information. The limitation of visual features makes inspection systems exclude most object recognition methodologies that are based on sophisticated texture and shape features [4].

This paper presents an online AIS for vehicle body surface defects. The AIS comprises an image acquisition subsystem (IAS) and image processing subsystem. The IAS acquires gray automobile images for the surface of a vehicle body. After that, the automobile image is processed and possible defects are detected. In this paper, we focus on three key techniques of AIS: image preprocessing, defect binarization, pseudo-defect removal and classification. We propose image preprocessing methods to remove the specular highlight pixels and enhance the distinction between defects and background in a vehicle image, considering imbalanced illumination and specular highlight reflection property of vehicle surfaces. In addition, we put forward multi-scale defect binarization based on a Hessian matrix (DBHM) algorithm, which identifies possible defects by construction of defect filter based on the Hessian matrix over each acquisition of the image detection area. Finally, we come up with pseudo-defect removal and classification on account of the noise-sensitive characteristics of Hessian filters and the existence of pseudo-defects. AIS has the following advantages:

1) Image preprocessing is a data fusion and image enhancement step. Therefore, it is able to eliminate the specular highlight reflection of raw images. In addition, it can greatly reduce the variation in the background of a vehicle image.
2) DBHM combined with pseudo-defect removal and classification is robust to noise and easy to identify tiny scratches or dents with a width of 0.5 mm, because DBHM relies on the local distribution and features of intensities, rather than the global characteristics used in most thresholding algorithms [5,6,7,8].
3) AIS has the characteristics of fast speed, high precision, low cost and nondestructive features. Under our experimental setup, VIS is able to be in real time with a speed of 1 min 50 s/vehicle, which is greatly faster than the manual inspection of check-man (10–15 min).

The rest of paper is organized as follows: in Section 2, related work on automatic defect inspection of vehicles is introduced. Then, the overall system and proposed approach are respectively described in detail in Section 3 and Section 4. Afterwards, Section 5 presents the experimental results. Finally, discussion and conclusions are provided in Section 6.

## 2. Related Work

Defect detection on car bodies usually adopts an inspection program flow, which can perform image acquisition, fusion by collecting the images of car bodies, image processing and classification to detect and distinguish millimeter-sized defects. Various vehicle inspection systems and techniques have been introduced with the aim to detect surface defects.

A commercial and very successful system was developed and installed in Ford Motor Company factories all around the world. This system, described in [9], uses a moving structure made up of several light bars (high-frequency fluorescent tubes) and a set of cameras in fixed positions around the stationary car body, and is able to detect millimetric defects of 0.3 mm diameter or greater with different shapes which were very hard to detect, thus improving the quality of the manual inspection carried out until that point. Thenceforth, Molina et al. [2] introduced a novel approach using deflectometry- and vision-based technologies at industrial automatic quality control system named QEyeTunnel in the production line at the Mercedes-Benz factory in Vitoria, Spain. The authors present that the inspection system satisfies cycle time production requirements (around 30 s per car). A new image fusion pre-processing algorithm is proposed to enhance the contrast between pixels with a low level of intensity (indicating the presence of defects) based on rendering equation [10,11] in computer graphics; Then a post-processing step with an image background extraction approach based on a local directional blurring method and a modified image contrast enhancement, which enables detection of larger defects in the entire illuminated area with a multi-level structure, given that each level identifies defects of different sizes.

Fan et al. [12] demonstrated a closed indirect diffusion-lighting system with the pattern-light and full-light mode by the lighting semi-permeable membrane to overcome the strong reflection of the car’s body. Then, a smooth band-pass filter based on frequency domain analysis processing technology is applied to extract the defect information on the panels from the used cars. In order to improve the accuracy of the result and remove the pseudo-defects, the area size is employed by the authors as the parameter to filter out the noise and achieve the segmentation of flaw for the reason that compared with flaws, noise is always some isolated points which just occupy a few pixels, but by filtering noise only by the area information of the noise (one important feature, but not comprehensive enough), it is possible to mistakenly filter some small-area pixels which originally were a defect. Kamani et al. [13,14] located defect regions by using a joint distribution of local binary variance pattern (LBP) and rotation invariant measure of local variance (VAR) operators, and then the detected defects are classified into various defect types using Bayesian and support vector machine classifiers. Chung et al. [15] implemented a optical 3D scanner and visualization system to find defect regions from the curvature map of unpainted car body outer panels. Leon et al. [16] presented new strategies to inspect specular and painted surfaces. This method classified defects into three classes: tolerable defects, removable defects and defects that lead to the rejection of the inspected car body part with 100% classification accuracy. Borsu et al. [17] used 3D reconstruction of the surface profile of the panel to detect deformation and the positions of the surface defects are provided to a robotic marking station that handles pose and motion estimation of the part on an assembly line using passive vision. Xiong et al. [18] proposed a 3D laser profiling system which integrates a laser scanner, odometer, inertial measurement unit and global position system to capture the rail surface profile data. The candidate defective area is located by comparing the deviation of registered rail surface profile and the standard model with the given threshold. Then, the candidate defect points are merged into candidate defect regions using the K-means clustering and the candidate defect regions are classified by a decision tree classifier.

In addition to the traditional detection methods mentioned above, learning-based methods have also been investigated for surface crack detection. Qu et al. [19] proposed a bridge cracks detection algorithm by using a modified active contour model and greedy search-based support vector machine. Zhang et al. [20] used a deep convolutional neural network to conduct automatic detection of pavement cracks on a data set of 500 images collected by a low-cost smart phone. Fully convolutional neural networks were studied to infer cracks of nuclear power plants using multi-view images by Schmugge et al. [21]. Recently, Zou et al. [22] proposed a novel end-to-end trainable deep convolutional neural network called DeepCrack for automatic pavement crack detection by learning high-level features for crack representation. In this method, multi-scale deep convolutional features learned at hierarchical convolutional stages are fused together to capture the line structures. Many other methods were also proposed for crack detection, e.g., the saliency detection method [23], the structure analysis methods by using the minimal spanning tree [24] and the random structure forest [25].

## 3. Overall System

**Figure 2:**
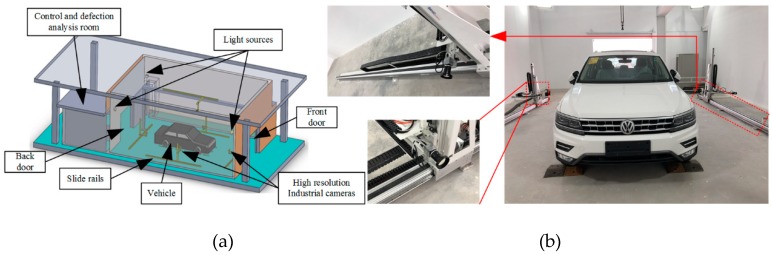
Laboratory of vehicle automatic inspection system. (**a**) Sketch of the vehicle inspection laboratory. (**b**) The actual experimental scene. The cameral is installed at 3-axis robot aluminum arms which are motor-driven by slide rails.

In order to eliminate the influence of natural light irradiated on the vehicle body, the detection process will be carried out in a closed laboratory environment shown in Figure 2. Five plane-array CCD cameras are mounted on motor-driven slide guides to achieve fixed-point acquisition of vehicle body images. A set of images is acquired during cameras sweeping the vehicle body ensuring complete cover of the object to be inspected, see Figure 2b.

The number of images depends on the size of the vehicle and the resolution of acquisition of images by the camera. These images are transmitted to the control and defection analysis room for further detection and classification. The control and defection analysis room is also used to control the light sources, the camera shooting and the operation of the guide rail motors.

### 3.1. AIS

**Figure 3:**
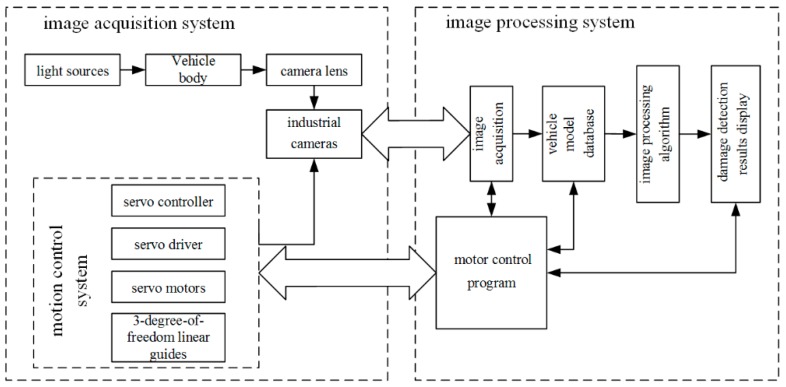
Overall architecture of the vehicle automatic inspection system.

The overall architecture of vehicle automatic inspection system is depicted in Figure 3. The automatic inspection system of appearance defect for automobile mainly includes two parts, namely, image acquisition subsystem (hardware) and image processing subsystem (software). The hardware system mainly includes light sources, industrial cameras, camera lens, slide rails, servo controller, servo driver and servo motors. The slide rails, servo controller, servo driver and servo motors constitute the motion control system. Software system mainly includes image acquisition, vehicle model database, image processing algorithms, damage detection results display and motor control program module.

### 3.2. Cameral Resolution

The key component of the proposed system is a GCP4241 GigE Vision plane-array CCD camera (SMARTEK Vision, Cakovec, Croatia) with 12 megapixels (4240 × 2824) at a frame rate of 9 fps which is the perfect for moving object inspection, low-light and outdoor applications or in high-speed automation with very short exposure times. Full Gigabit Ethernet bandwidth based on the proven GigE Vision standard data interface is utilized to support high resolution image transmission. With a C-10MP-4/3-12mm lens (Foshan HuaGuo Optical Co., Ltd., Guangdong, China) with a shooting distance of 1.5 m, the resolution of the camera can reach 72 dpi (0.35 mm/pixel) which meets the accuracy requirements for detecting defects above 0.5 mm in size.

### 3.3. Framework of Image Processing Subsystem

**Figure 4:**
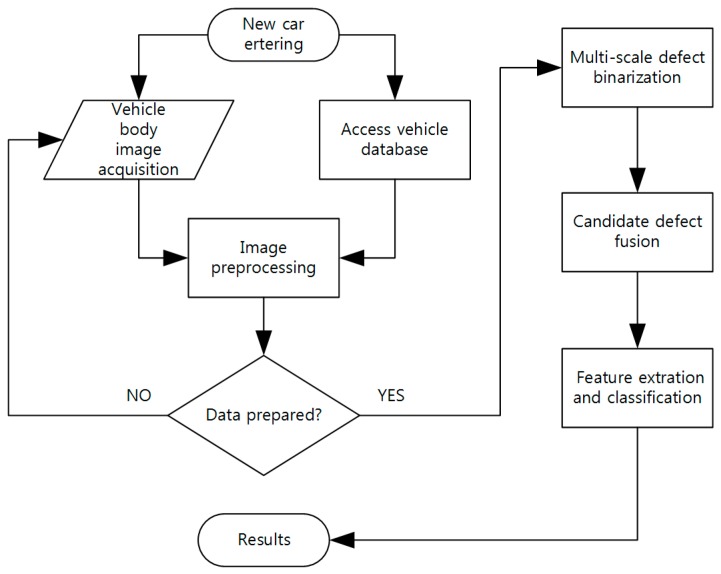
Diagram of image processing subsystem.

After a vehicle body image is generated by the IAS, an image processing subsystem is proposed to detect possible defects in the image. This subsystem includes three main modules: image preprocessing, defect binarization and pseudo-defect remove and classification. Figure 4 shows the diagram of the subsystem:

1) Image preprocessing: Defects are easily hidden or confused in vehicle images because of illumination inequality and the variation of reflection property of vehicle surfaces, so image preprocessing is a necessary procedure to highlight defects area from the image background. Effective image preprocessing process can improve the efficiency and classification accuracy of subsequent algorithms.
2) Defect binarization: Defect binarization is the process of extracting candidate defect areas from a vehicle body. The second-order information of defect region can be extracted by DBHM method, and the detection rate of micro-defects can be improved by binarizing the possible defect regions.
3) Feature extration and classification: A defect binarization image comprises three types of pixels: defects, background and pseudo-defect generated by noise of the preprocessed image. Due to the influence of noise and image quality, there are cases of pseudo-defects after binarization of defects. Through feature extraction of candidate feature regions, we can classify defects while excluding some pseudo-authorities, thereby improving the detection accuracy of AIS.

## 4. Proposed Approach

### 4.1. Image Preprocessing

The image formation process consists of two steps: optical reflection and photoelectric conversion. In the first step, the illumination model converts the physical properties such as the reflectivity of the vehicle body surface into the reflected light intensity. The second step converts the reflected light intensity into a digital image of the vehicle body through a camera lens and an analog-to-digital converter (ADC or A/D). Based on the understanding of the physical properties of light, the Phong illumination model (PIM) is a widely used illumination physics model. PIM was first proposed by Phong [26] and improved by Whitted [27]. Its main structure consists of ambient term, diffuse term and specular term:

(1) $$R_{p}=I_{p}^{amb}+\sum_{m\in lights}\left(k_{d}^{dif}\left(\vec{L_{m}}\cdot \vec{N}\right)I_{m,d}^{l}+k_{d}^{spe}\left(\vec{R_{m}}\cdot \vec{V}\right)I_{m,d}^{l}\right)$$

where

*R_p_* reflected intensity at position *p*;
$I_{p}^{amb}$ reflection intensity of ambient light at position *p*;
*m* serial number of light sources
$I_{m,d}^{l}$ Light intensity of the *m*-(th) light source
$k_{d}^{dif}$ diffuse reflection coefficient;
$k_{d}^{spe}$ specular reflection coefficient;
$\vec{N}$ normal direction of position *p*;
$\vec{V}$ direction of view (usually the direction of the camera);
${\vec{L}}_{m}$ direction of the *m*-(th) light source;
${\vec{R}}_{m}$ perfect mirror direction.

For multiple point light sources, the PIM sums up diffuse term and specular term simultaneously for each individual light source [26]. In the second step, the photoelectric process converts the reflected light intensity *R_p_* into a digital image *I_p_* of the vehicle body through a camera lens and an analog-to-digital converter (ADC or A/D). This transformation can be expressed by a linear formula:

(2) $$I_{p}=\alpha \cdot R_{p}+\beta$$

where *α* and *β* are constant parameters determined by camera setup [27].

The quality of automobile body image acquisition determines the results of defect detection in subsequent image processing. The two formulas of the body imaging model provide guidance for automotive appearance defect detection experiments:

1) Using indirect diffused light pattern and increasing the incident angle of the light source can reduce or remove the specular reflection item in the body image to obtain a uniform image of the vehicle body;
2) The method of image superposition can effectively remove the Gaussian noise in the initial image (this experiment uses the output image after superimposing six consecutive image frames as the actual image to be detected).

### 4.2. Candidate Defect Binarization

The Hessian matrix is a common method for extracting image features by high-order differential [28]. The Hessian method regards the direction of the second-order directional derivative with the largest module as the direction perpendicular to the image feature, and the direction perpendicular to it as the direction parallel to the image feature. For a linear model with Gauss function, it can be expressed by the second derivative with larger absolute value orthogonal to the straight line and the second derivative with smaller absolute value along the line. This is exactly the geometric meaning of the two eigenvalues of the two-dimensional Hessian matrix. In the process of binarization of candidate defects, we conceive the binarization of defects as a filtering process that searches for geometric structures which can be regarded as point or linear defects. Because the size of defects is different, it is important to introduce measurement scales that vary within a certain range.

The local behavior of an image *I* can be approximated to its second-order Taylor expansion [29]. Thus, image *I* in the neighborhood of point **x***_o_* can be expressed as:

(3) $$I\left(x_{o}+\delta x_{o},s\right)\approx I\left(x_{o},s\right)+\delta {x_{o}}^{T}\nabla_{o,s}+\delta {x_{o}}^{T}{Η}_{o,s}\delta x_{o}$$

This expansion approximates the structure of the image up to second order. ∇_*o*,*s*_ and **H**_*o*,*s*_ are respectively the gradient vector and Hessian matrix of the image in position **x***_o_* at scale *s*. According to the concept of linear scale space theory, differentiation is defined as a convolution with derivatives of Gaussians:

(4) $$\frac{\partial }{\partial x}I\left(x,s\right)=I\left(x\right)*\frac{\partial }{\partial x}G\left(x,s\right)$$

where the two-dimensional Gaussian is defined as:

(5) $$G\left(x,s\right)=\frac{1}{2\pi s^{2}}e^{-\frac{x^{2}+y^{2}}{2s^{2}}}$$

Assume that the Hessian matrix is expressed as $H_{o,s}=\left[\begin{array}{cc} H_{xx} & H_{xy}\\ H_{yx} & H_{yy} \end{array}\right],\;(H_{xy}=H_{yx}$). Eigenvalues of the Hessian matrix can be calculated as:

(6) $$\lambda_{1}=\frac{1}{2}\left(H_{xx}+H_{yy}+\sqrt{{\left(H_{xx}-H_{yy}\right)}^{2}+4H_{xy}}\right)$$

(7) $$\lambda_{2}=\frac{1}{2}\left(H_{xx}+H_{yy}-\sqrt{{\left(H_{xx}-H_{yy}\right)}^{2}+4H_{xy}}\right)$$

The eigenvalue of the Hessian matrix satisfies the case of $|\lambda_{1}|\gg 0,|\lambda_{2}|\gg 0$ or $|\lambda_{1}|\gg |\lambda_{2}|,|\lambda_{2}|\approx 0$ at the location where a body defect exists. Thus, a two-dimensional candidate defect enhanced image is defined as:

(8) $$e=\{\begin{array}{l} 0,\;f\left(x,y\right)<f_{L}\;\mathrm{or}\;f\left(x,y\right)>f_{H}\\ \frac{1}{2}\left(H_{xx}+H_{yy}+\sqrt{{\left(H_{xx}-H_{yy}\right)}^{2}+4H_{xy}}\right)-th,\;\mathrm{otherwise} \end{array}$$

where *th* is a constant threshold value, which is used to enhance the possible defective regions. The background noise in the vehicle body diagram is directly removed by *f*(*x*,*y*) < *f_L_* and *f*(*x*,*y*) > *f_H_*, and the subsequent mask to background interference operation is reduced.

**Figure 5:**
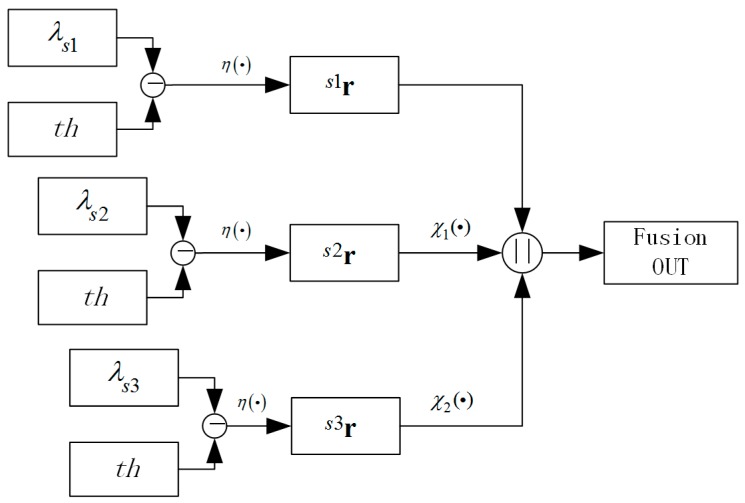
Three level scales DBHM binary fusion method.

For body defect structural elements, the output of defect region in enhanced image is maximized only when the scale factor *s* best matches the actual width of the defect, so we introduce a three level scales detection method as shown in Figure 5. By performing a global thresholding of enhanced images *^s^*^1^**e**, *^s^*^2^**e**, *^s^*^3^**e** in different scales *s*_1_, *s*_2_, *s*_3_ (usually *s*_1_ = 1), the middle result images can be obtained as *^s^*^1^**r**, *^s^*^2^**r**, *^s^*^3^**r**, the presentations of which are defined as *^s^*^1^**r** ∈ {0,1}**^X^**, *^s^*^2^**r** ∈ {0,1}**^Y^**, *^s^*^3^**r** ∈ {0,1}**^Z^**. $X=Z_{m}\times Z_{n}=\left\{x:=\left(x,y\right)\in Z^{2}|0\leq x\leq m-1,0\leq y\leq n-1\right\}$, where *m* and *n* are the number of columns and rows of the image, $Y=Z_{\left[m\times \frac{s2}{s1}\right]}\times Z_{\left[n\times \frac{s3}{s1}\right]}$, and $Z=Z_{\left[m\times \frac{s3}{s1}\right]}\times Z_{\left[n\times \frac{s3}{s1}\right]}$. The binary pattern images can be obtained as follows:

(9) $$\begin{array}{l} {}^{s1}r=\eta \left({}^{s1}e\right)\\ {}^{s2}r=\eta \left({}^{s2}e\right)\\ {}^{s3}r=\eta \left({}^{s3}e\right) \end{array}$$

where $\eta =\{\begin{array}{c} 1\;\mathrm{if}\;e>0\\ 0\;\mathrm{if}\;e<0 \end{array}$. By iterating the scale factor, the images at different scales are obtained, and the union of the defect regions is taken as the final candidate defect image:

(10) $$OUT:={}^{s1}r\|\chi_{1}\left({}^{s2}r\right)\|\chi_{2}\left({}^{s3}r\right)$$

where $\chi_{1}:R^{Y}\to R^{X},\;\chi_{2}:R^{Z}\to R^{X}$ are the up-scaling operators with **Z** ⊂ **Y** ⊂ X. ‖ is the bitwise or operator.

### 4.3. Feature Extraction of Candidate Defects

Since the candidate defects filtered by the Hessian matrix are easily affected by image noise, features of the defective region like shape, size and divergence are introduced to generate the feature vectors for training and remove pseudo-defects produced by image noise. In order to quantify the shape and size of the defect region, five common image features is utilized: area, major length, minor length, aspect ratio of minimum bounding rectangle, thinness ratio, and to distinguish the real defects from the pseudo defect regions, five statistical characteristics of the candidate region are utilized: mean pixel value, standard deviation, mean value of regional divergence, standard deviation of regional divergence and maximum value of regional divergence. The details of the defect feature are described as follows.

Area is the actual number of pixels in the candidate defect region, which is proportional to the actual area of the defect on the vehicle body.

The major length and minor length of minimum-area-rectangle (MAR) specify the major and minor side length (in pixels) of the minimum rotational circumscribed rectangle that contains all the point sets in the defective region. The aspect ratio of MAR is the ratio of the major length and minor length of the smallest circumscribed rectangle in the defect region.

Thinness ratio [30] describes the similarity between the defect area and the circle which can be calculated from the perimeter and area. The equation for thinness ratio is defined as follows:

(11) $$T=4\pi \frac{A}{P^{2}}$$

where *A* is the area and *P* is the perimeter of the defect which is defined as the total pixels that constitute the edge of the defect. As the defect becomes thinner and thinner, the perimeter becomes larger and larger relative to the area and the ratio decreases so the roundness decreases. As the shape of defect becomes closer to a circle, this ratio become closer to 1.

Mean pixel value and standard deviation of candidate defect regions are utilized to eliminate pseudo-defect areas. In addition to mean and standard deviation, we also propose the concept of divergence in feature extraction. From the point of view of energy diffusion, the divergence of gradient image (two-dimensional vector field) is the extent to which the vector field flow behaves like a source at a given point. It is a local measure of its “outgoingness”—the extent to which there is more of some quantity exiting an infinitesimal region of space than entering it [31]. If the divergence is positive, it means that the vector fields are scattered outward. If it is negative, it means that the vector fields are concentrated inward. That is to say, divergence indicates the direction of the flow of the vector, and the greater the degree of the flow, the faster the divergence, the greater the corresponding divergence value. The divergence of a vector field is defined as follows:

(12) $$\mathrm{div}F:=\underset{V\to 0}{\lim}\frac{1}{V}\underset{\partial V}{∯}F\cdot dS$$

In the 2-dimensional Descartes coordinate system, the divergence of image gradient field *grad*(**I**) = *U***i** + *V***j** is defined as the scalar-valued function:

(13) $$\mathrm{div}\left(grad\left(I\right)\right):=\nabla \cdot grad\left(I\right)=\left(\frac{\partial }{\partial x},\frac{\partial }{\partial y}\right)\cdot \left(U,V\right)=\frac{\partial U}{\partial x}+\frac{\partial V}{\partial y}$$

where **i**, **j** are the corresponding basis of the unit vectors, *grad*(**I**) is the gradient of image **I**. Although expressed in terms of coordinates, the result is invariant under rotations, as the physical interpretation suggests. Thus, the mean value, standard deviation and maximum value of regional divergence also have the properties of translation and rotation invariance.

### 4.4. Defect Classification

After the feature of candidate defects been extracted, we employ SVM [32] to classify the candidate defects into three types: pseudo-defect, dents and scratches. SVM first project the data points into a feature space by kernel function, and then perform linear classification in the feature space. A typical mathematical formulation is to solve the following convex optimization problem:

(14) $$\begin{array}{l} \min_{\omega ,b,\xi_{i}}\frac{1}{2}\omega^{T}\omega +C\sum_{i}\xi_{i}\\ s.t.\;y_{i}\left(\omega^{T}\phi \left(x_{i}\right)+b\right)\geq 1-\xi_{i},\xi_{i}\geq 0 \end{array}$$

where **x***_i_* are the data samples, *y_i_* are the labels, *φ*(·) is the kernel function, and *C* is the penalty coefficient. Common kernel functions include linear kernels: $\phi {\left(x_{i}\right)}^{T}\phi \left(x_{j}\right)=x_{i}^{T}x_{j}$, and radial basis function (RBF) kernels: $\phi {\left(x_{i}\right)}^{T}\phi \left(x_{j}\right)=\exp\left(-\gamma {\left\|x_{i}-x_{j}\right\|}_{2}^{2}\right)$, where *γ* is a positive hyperparameter. The candidate Defects are divided into training and test sets using LIBSVM [33] to perform training and testing.

## 5. Experiments and Results

The AIS was jointly developed by the Nanjing University of Aeronautics and Astronautics (Nanjing, China) and the Shanghai Together International Logistics Co., Ltd. (Shanghai, China). All the experiments in this paper are built with the OpenCV C++ module in the Windows 7 operating system. PC desktop hardware configuration: dual-core Xeon SP CPU, 32 G memory, P4000 8 G unique. The body image was collected by a GCP4241C industrial camera of the Smartek Vision Company (Cakovec, Croatia). The images have a 4240 × 2824 size in 8-bit BMP format and 0.35 mm × 0.35 mm resolution. A data set with 96 images is constructed to evaluate the system performance, where each image contains at least five defects. As seen in Section 3 and Section 4, the defect detection algorithm proposed in this paper consists of three phases: an image preprocessing step, in which image superposition and region of interest extraction are proposed, a candidate defect binarization step, in which multi-scale fusion of largest of eigenvalue of Hessian matrix method is proposed, and a feature extraction and classification step, in which the eight features of candidate defects are extracted and these candidate defects are classified into scratches, dents and pseudo-defects.

### 5.1. Preprocessing Step: Image Superposition and Region of Interest Extration

**Figure 6:**
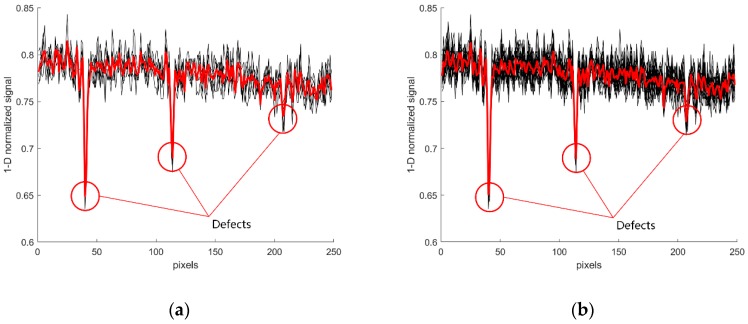
Image superposition with different number of sheets (**a**) superposition of six vehicle images (**b**) superposition of 20 vehicle images.

In Figure 6, the red one-dimensional signal is the result of the average value of multiple images. By comparing the results of image Figure 6a,b, we can draw the conclusion that the superposition results of six images are similar to those of 20 images, which can effectively reduce the Gauss noise in the image. At the same time, the computational complexity of six images superposition is less than that of 20 images superposition. Therefore, the follow-up experimental pictures all use six image superimposed pictures.

Although the photoelectric distance sensor installed on the boom can accurately determine the position of the camera, the manual parking way used may cause a certain deviation in the spatial coordinate position of the vehicle image. Therefore, before the candidate defect is extracted, the region of interest (ROI) region is extracted using image registration. Image registration is obtained by feature point extraction, feature point description and feature point matching, and then the spatial transformation matrix estimation of two similar images is obtained. Compared with the traditional SIFT method [34], the SURF method [35] uses a small amount of data and has a short calculation time, therefore, the SURF registration method is adopted in this paper.

**Figure 7:**
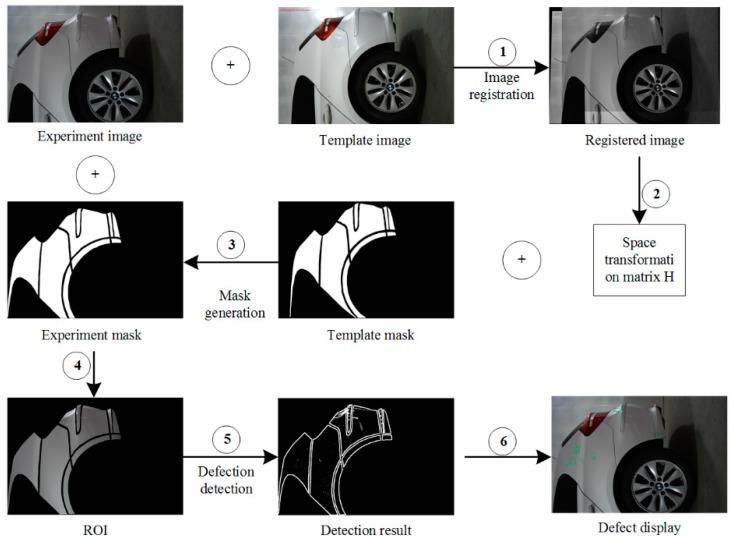
Examples of detection of ROI defects detection procedure.

When a new body is ready to be introduced into the inspection system, the checker manually selects the test vehicle type on the PC software detection interface and the database provides all the necessary body-related data (e.g., model, color, template, mask, etc.) The complete body defect detection process is illustrated in Figure 7. Firstly, the spatial transformation matrix H is obtained from the experimental image and template image by the registration principle (steps ① and ②). Secondly, the template mask corresponding to the template image is read by the database, and the mask of the image to be detected is obtained by interpolating the space transformation matrix H, that is, the area to be detected (ROI) of the experimental image (steps ③ and ④). Finally, the actual defect area is obtained by the proposed defect detection algorithm and marked with green box on the experimental map (steps ⑤ and ⑥).

**Figure 8:**
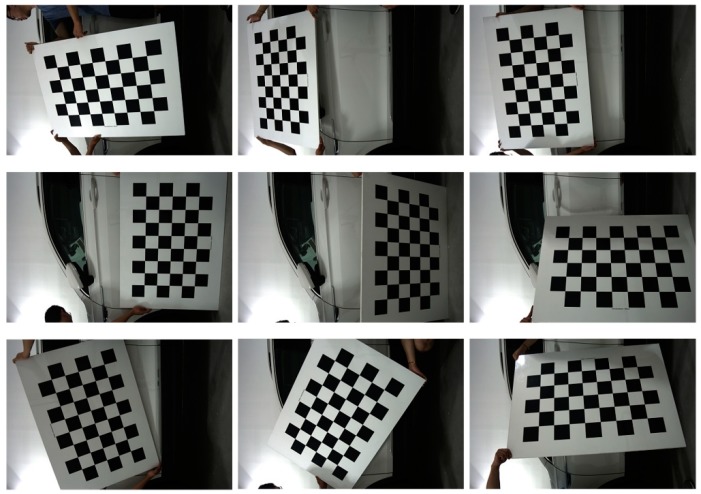
Camera calibration using a 6 × 9 × 100 mm^3^ chessboard.

In order to improve the accuracy of image registration, we introduce three steps before registration which are camera calibration (Figure 8), edge detection and area opening. This means that the registration process of ROI extraction is not performed directly on gray scale vehicle images, but on the edge images after area opening operations (excluding noise pixels). The lens distortion of the camera has a great influence on the accuracy of image registration. Through experiments, we find that the edge pixel error of the calibrated camera is decreased from more than a dozen pixels to 2–5 pixels compared with the uncalibrated camera, which meets the requirement of body detection area extraction.

### 5.2. Candidate Defect Binarization: Case Study and Multi-Scale Fusion Method

**Figure 9:**
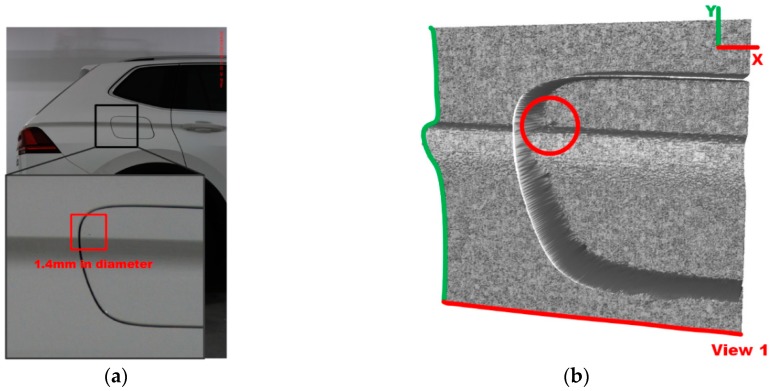

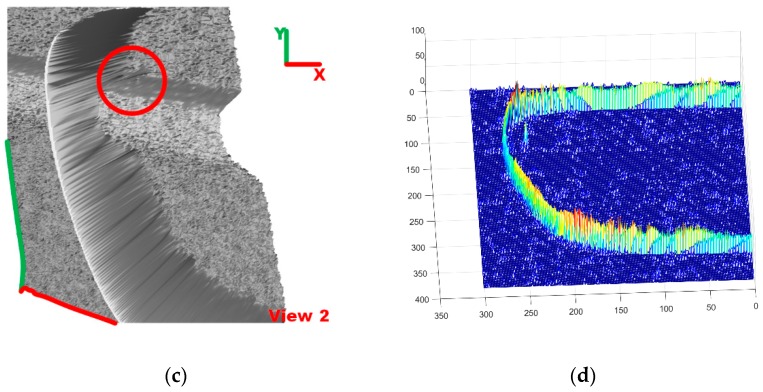
Case study: evaluation of the proposed algorithm dealing with a defect close to fuel tank cap (**a**) Detection region on fuel tank cap; (**b**) 3D representation of the magnified region (view1); (**c**) 3D representation of the magnified region (view2); (**d**) 3D map of *λ*_1_ in DBHM.

Figure 9 shows an example of defect detection for a dent with a diameter of 1.4 mm (only occupying nine pixels) near the style line on the fuel tank cap. From the 3D representation of the detection region in Figure 9b,c, it can be seen that the location of the defect is very close to the style line and the edge of the fuel tank cap.

With a global threshold (T = 16) set by the binarization filter in Equations (8) and (9), we can distinguish between noise and defect and thus identify the defect on the surface as a result which is shown in Figure 9d.

**Figure 10:**
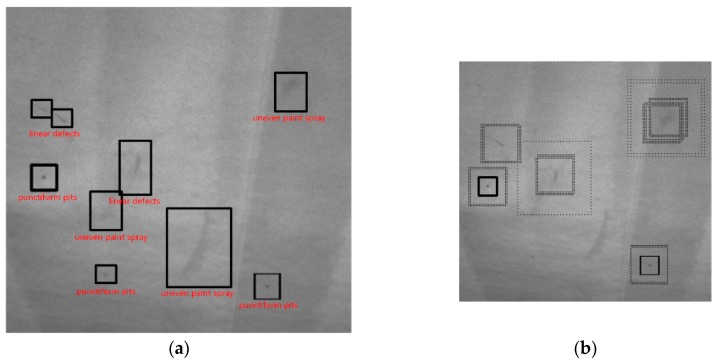

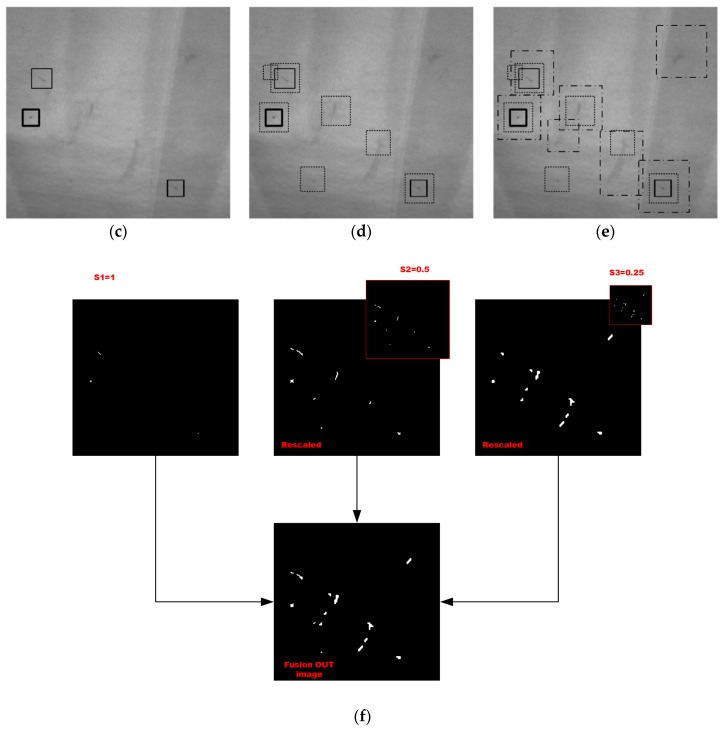
Illustration of three level scales detection process (**a**) ground truth of surface defects (**b**) detection result of enhancement background subtraction of three-level detection method proposed by Jaime Molina [2] (**c**) our first scale level detection represented by a small solid-line box, (**d**) our second scale level detection represented by a broken-line box (**e**) our third scale level detection by a large dotted-line box (**f**) our final fusion candidate defect image.

In Figure 10, the picture used for experiments is acquired from an automated vision-based quality control inspection system named QEyeTunnel in Molina’s paper [2]. Their inspection system consists of two parts: an external fixed structure where a determined number of cameras are optimally placed in order to see the entire surface of the body to be analyzed, as well as a moving internal structure similar to a scanning machine which houses curved screens known as ‘sectors’ which act as the light sources that project the illumination patterns over the body surface. Figure 10a shows nine defects of automobile surface after spraying paint on automobile production line. This vehicle body image contains punctiform pits, linear defects and uneven paint spray that are difficult for the human eye to distinguish.

In our method, the most obvious line and point scratches are extracted in the first scale level detection (*s*_1_ = 1). Defects of uneven paint spray which are difficult for human eye to distinguish are detected in second and third scale level detection (*s*_2_ = 0.5, *s*_3_ = 0.25). After the second and third level images are rescaled to the first level scale of detection result, the final image result detects all types of defects which is merged by the bitwise or operator using the three level rescaled results.

By comparing the graphs of Figure 10b,e, we can conclude that, for the spot-like, linear and unevenly sprayed surface defects that are difficult to detect in the human eye, our proposed multi-scale Hessian eigenvalue method can still detect these defects.

### 5.3. Feature Extration and Classification of Candidate Defect Region

We extract 96 images from the image acquisition system. Then we search the connected components form the fused binary pattern of candidate defect regions and label the defect types manually. We then split it into a training subset and a test subset, with the training subset containing 70% of the data and the test subset containing the remainder. Grid search with fivefold cross-validation is performed on the training subset, and report generalization performance on the test subset. Specifically, for linear kernel, we try C with C = 2^−5^, 2^−4^, …, 2^15^. The best hyperparameters for the linear kernel are shown in Table 1.

From Table 1, we can see that the classification accuracy of scratches is high which is up to 97.1%, but the relative accuracy of pits, 95.6%, is low. A possible reason is that the average pixel area of dents usually occupies only a single-digit number of pixels. The occurrence of these misclassified pit defects may be due to dust falling on the surface of the vehicle body during transportation and false defects caused by image noise. 

**Figure 11:**
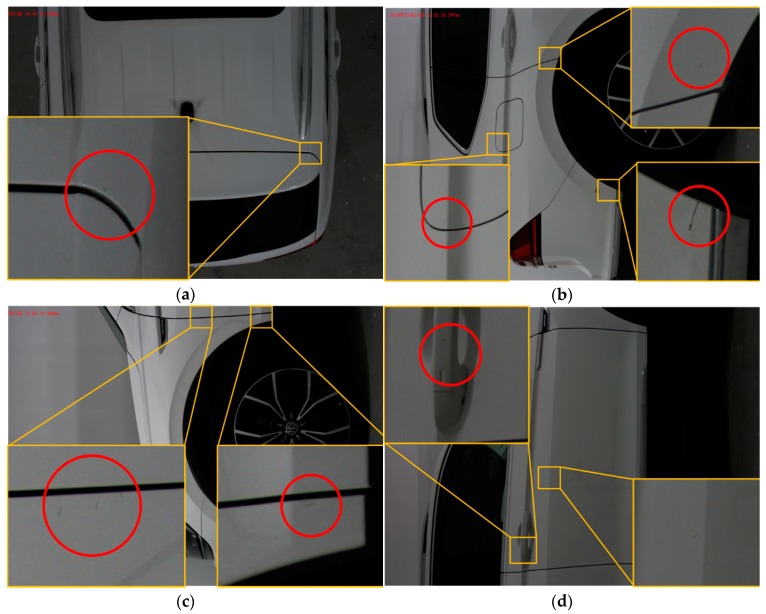
Set of detection examples showing the detection of defects on different position of car bodies (**a**) one defect near edge of body trunk cover on the roof of the car, (**b**) two defects near the edge of the fender surface (**c**) three defects, one on the fuel tank cover two near the edge of the fender surface (**d**) two defects, one on the handle, one near the style line.

To demonstrate the performance of the defect detection algorithm presented in this paper, a selection of results obtained on the production line with different parts and car bodies is shown in Figure 11. In some of these, detections are observed close to or on style lines and surface edges, as in Figure 11a–c. In addition, it is possible to see detections carried out in concave areas such as the handle, Figure 11d.

## 6. Conclusions and Future Work

In this paper, we have developed an AIS for detection of discrete surface defects of vehicle bodies. The proposed AIS has the ability to detect defects which are the located in or close to style lines, edges and handles, thus demonstrating the detection power of our system. In particular, we put forward a three-level scales DBHM detection method for extracting of candidate defects on the vehicle surface. This method distinguishes the defect location information and binarizes the candidate defects at three different scales by comparing the second-order features of the background from the image defects. This allows defect detection in concave areas or those with abrupt changes in the surface near style lines, edges and corners, provided that the area is well lit. Three level scales detection process is proposed to extract defects that are hard to distinguish from human eyes by fusing the multi-layer results under the same scale. In addition, we propose the feature extraction method of candidate defects and divide defects into three categories (pseudo-defects, dents, scratches) through linear SVM classifier. The experimental results demonstrate that AIS detects the dent defects with accuracy of 95.6% and scratch defects with accuracy of 97.1%. Furthermore, VIS is a very fast system with a speed of 1min50s/vehicle, which is faster than the manual inspection of check-man.

Although AIS is validated to be an effective system for detecting vehicle surface defects, it can be further improved in three aspects:

1) Deflectometry techniques [36] show promise in image defect contrast enhancement on specular surfaces. If the funds are sufficient, we will try this lighting method to collect the body images.
2) The precision of AIS could be improved if the selection of the parameter *th* for the multi-scale DBHM method could be more intelligent, although this has the risk of slowing down the detection speed. We will investigate adaptive selection approaches.
3) The detection speed of the AIS could be further increased if it is paralleled using parallel computing techniques, such as general-purpose computation on graphics processing units. The five plane-array CCD cameras collect images of the five sides of the automobile synchronously, so the AIS has the potential to be paralleled and accelerated.

## Figures and Tables

**Table 1:** Optimal hyperparameters and accuracy for linear kernel.

Defect Type	C	Training Accuracy ± Standard Deviation	Testing Accuracy ± Standard Deviation
Pseudo-defects	0.015625	98.2 ± 0.9%	96.0% ± 1.8%
Dents	0.015625	96.5 ± 2.6%	95.6% ± 3.4%
Scratches	0.015625	98.6 ± 1.3%	97.1% ± 1.6%
